# Role of NK cells in cord blood transplantation and their enhancement by the missing ligand effect of the killer-immunoglobulin like receptor

**DOI:** 10.3389/fgene.2022.1041468

**Published:** 2022-10-18

**Authors:** Hisayuki Yokoyama

**Affiliations:** Department of Hematology, Tohoku University Graduate School of Medicine, Sendai, Japan

**Keywords:** NK cell, cord blood transplantation (CBT), killer cell immunoglobulin-like receptor (KIR), GvHD prophylaxis, cytomegalovirus

## Abstract

Natural killer (NK) cells are the first lymphocytes reconstituted after allogenic hematopoietic stem cell transplantation (HSCT). Especially, in cord blood transplantation (CBT), the increase in the number of NK cells is sustained for a long period. Although there are conflicting results, many studies show that early reconstitution of NK cells is associated with favorable CBT outcomes, suggesting that maximizing NK cell functions could improve the CBT outcome. Killer immunoglobulin-like receptors (KIRs) include inhibitory and stimulatory receptors, which can regulate NK-cell activity. Because some of the KIRs have HLA class I as their ligand, the KIR—ligand interaction on NK cells can be lost in some cases of CBT, which results in the activation of NK cells and alters HSCT outcome. Thus, effects of KIR–ligand mismatch under various conditions have been widely examined; however, the results have been controversial. Among such studies, those using the largest number of CBTs showed that HLA—C2 (KIR2DL1—ligand) mismatches have a favorable effect on the relapse rate and overall survival only when the CBT used methotrexate for graft-versus-host disease prophylaxis. Another study suggested that KIR—ligand mismatch is involved in reducing the relapse of acute myeloid leukemia, mediated by reactivation of cytomegalovirus. These results indicate that activation of NK cells by KIR—ligand mismatch may have favorable effects on CBT outcomes and could help enhance the NK-cell function.

## Introduction

Allogeneic hematopoietic stem cell transplantation (HSCT) is a widely used treatment for relapsed or refractory hematological malignancies. HSCT outcomes have gradually been improving, however they are not yet satisfactory (Auletta et al., 2021), and the main reason for the failure of HSCT is disease relapse (Auletta et al., 2021). Various attempts have been made to reduce relapses after HSCT (Mawad et al., 2013; Yafour et al., 2017; Lee et al., 2019; Kreidieh et al., 2022). In acute myeloid leukemia (AML), the effects of the prophylactic use of donor lymphocyte infusions (DLI) or small molecule compounds, such as tyrosine kinase inhibitors and hypomethylating agents, have been examined and their efficacies have been reported (Burchert et al., 2020; Wei et al., 2020; Xuan et al., 2020). Although these treatments considerably improved HSCT outcomes, their efficacy is limited for some patients and cannot sufficiently reduce the relapse, warranting further development.

Recently, there have been remarkable developments in the treatment of hematological malignancies, mediated by immunological mechanisms, such as bispecific antibodies (Ma et al., 2021) and chimeric antigen receptor T cells (June and Sadelain, 2018). HSCT is one of the immunotherapies that utilizes the strong immune reaction of donor lymphocytes against a patient’s tumor cells. This reaction is called the graft-versus-leukemia effect (GVL effect) (Dickinson et al., 2017; Barisic and Childs, 2022). However, the GVL effect is closely associated with graft-versus-host disease (GVHD), which increases treatment-related mortality due to injury to normal organs (Negrin, 2015). To enhance the GVL effect, DLI has historically been used to treat or prevent post-transplant relapse, although it is associated with the risk of exacerbating GVHD and the benefits of DLI are still under investigation (de Lima et al., 2014; Orti et al., 2017). Under these circumstances, the use of natural killer (NK) cells has been suggested as another promising strategy to elicit GVL effects without exacerbating GVHD (Orti et al., 2017).

To date, the effects of NK cells on HSCT outcomes have mainly been evaluated with regard to two aspects: NK-cell reconstitution and potential interaction between receptors on NK cells and their ligands. Many studies have demonstrated that early NK-cell reconstitution after HSCT results in improved outcomes (Bühlmann et al., 2011; Minculescu et al., 2016; Bejanyan et al., 2018; Ando et al., 2020). The receptor–ligand interaction effect of NK cells after HSCT has predominantly been examined using models that assume an interaction between the killer-immunoglobulin like receptor (KIR) and its ligand (Ruggeri et al., 2007, 2002; Hsu et al., 2006; Cooley et al., 2009; Symons et al., 2010). Although there is some controversy, a few reports suggest that NK-cell activation mediated by KIR has favorable effects on HSCT. Such effects associated with NK cells have mostly been examined in bone marrow (BMT) or peripheral blood stem cell (PBSCT) transplantation, and studies on cord blood transplantation (CBT) are relatively scarce. However, previous studies have demonstrated fast and sustained increases in the number of NK cells and suppression of T-cell functions after CBT compared with that after BMT and PBSCT (Ando et al., 2020), suggesting that NK cells may play a more important role in CBT than in HSCT with other donor sources. This review, therefore, focuses on CBT and summarizes the available data on the role for NK cells.

## Effects of NK-cell reconstitution in CBT

The kinetics of NK-cell reconstitution have been examined in various types of HSCTs and compared among donor sources. The NK cells are the lymphocyte subset that is reconstituted most quickly after HSCT (Fry and Mackall, 2005; Ogonek et al., 2016). In CBT, NK-cell recovery at 1 month post-transplantation was almost the same or higher than in BMT and PBSCT. However, in CBT, the NK cells continue to increase and high NK-cell counts persist for approximately 1 year, whereas such predominance of NK cells is not noted in the BM or PBSCT (Rénard et al., 2011; Jacobson et al., 2012; Kanda et al., 2012; Bartelink et al., 2013; Bejanyan et al., 2018; Hattori et al., 2018; Ando et al., 2020). In contrast to the NK cells, the recovery of the T cells in CBT was delayed when compared to that in BMT or PBSCT. The reason for the sustained increase in the number of NK cells is not clear, but it may be associated with the slow T-cell recovery, in view of the fact that the presence of T cells impairs the function and proliferation of NK cells after HSCT (Lowe et al., 2003; Cooley et al., 2005). The NK cells that were reconstituted after CBT showed immature phenotypes that initially expressed NKG2A and bright CD56 (Nguyen et al., 2017). Approximately 3 months after CBT, mature phenotypes with CD16 and CD56dim expression were obtained, which have high cytolytic activity (Tanaka et al., 2009; Kanda et al., 2012; Ando et al., 2020).

The association between NK-cell recovery and HSCT outcomes has been examined in many studies (Dunbar et al., 2008; Bühlmann et al., 2011; Bartelink et al., 2013; Pical-Izard et al., 2015; Minculescu et al., 2016; Nguyen et al., 2017; Bejanyan et al., 2018; Hattori et al., 2018; Russo et al., 2018; Willem et al., 2019; Ando et al., 2020; Zhao et al., 2022). Among these studies, those including CBT cases are shown in Table 1 (Bartelink et al., 2013; Nguyen et al., 2017; Bejanyan et al., 2018; Hattori et al., 2018; Ando et al., 2020). In two of these studies, the effect of NK-cell recovery could not be detected, whereas in others, favorable impact on HSCT outcomes was observed. This discrepancy may be due to differences in patient background or in the transplantation methods. In addition, Ando et al. (2020) reported that only increases in the NK cell subpopulation, but not in the NK cell bulk population, has significant effects. These results, such as improvement in progression-free survival and relapse rate, suggest that NK cells are responsible for the antitumor effects during the period of reduced T cell function after CBT.

**TABLE 1:** Effects of early natural killer (NK)-cell recovery in the cohort that includes patients undergoing cord blood transplantation.

References	Author	N	HSCT year	Age	Disease (% of AML)	Donor source	Intensity of conditioning	ATG	GVHD prophylaxis	Effect of early NK-cell recovery
Bone Marrow Transplant; 52 (10): 1,428–1,435	Nguyen et al. (2017)	79	2007–2009	50 (33.7–58.2)	AML (100%)	CB	RIC	none	CSA/MMF	High NK-cell degranulation ability at 1 month was associated with better survival
Blood Adv. 2018; 2 (8): 909–922	Bejanyan et al. (2018)	157 (CBT 89)	2009–2014	61 (22–73)	Leukemia (66%)	PBSC (43%) CB (57%)	RIC	27%	CSA/MMF Sirolimus/MMF	No effect of NK-cell count at day 100 on OS, relapse, and TRM.
Biol Blood Marrow Transplant. 2018; 24 (9): 1841–1847	Hattori et al. (2018)	81 (CBT 48)	2003–2017	54 (18–71)	AML (48%)	BM (41%) PBSC (10%) CB (59%)	MAC (53%) RIC (47%)	none	CSA/MTX (3%)TAC/MTX (98%)	Early NK-cell recovery at day 21 was associated with OS, PFS, NRM, and incidence of infection
Blood Adv. 2020; 4 (2): 408–419	Ando et al. (2020)	310 (CBT 136)	2009–2017	51 (18–69)	AML (54%)	BM (38%) PBSC (18%) CB (44%)	MAC (36%) RIC (64%)	none	CSA/MTX (26%)TAC/MTX (74%)	Higher CD16^+^CD57^−^ NK-cell counts at day 100 correlated with lower disease relapse and favorable OS
Biol Blood Marrow Transplant. 2013; 19 (2): 305–13	Bartelink et al. (2013)	103 (CBT 36)	2004–2008	4.9 (0.1–21)	NA	BM/PBSC (65%) CB (35%)	NA	35% only for CBT	CSA (34%)CSA/MTX (31%) CSA/steroid (35%)	No effect of NK-cell count between day 0 and 90 on mortality

N, number of patients; CBT, cord blood transplantation; HSCT, hematopoietic stem cell transplantation; AML, acute myeloid leukemia; CB, cord blood; PBSC, peripheral blood stem cell; BM, bone marrow; RIC, reduced intensity conditioning; MAC, myeloablative conditioning; NA, not available; GVHD, graft-versus-host disease; CSA, cyclosporine A; MMF, mycophenolate mofetil; MTX, methotrexate; TAC, tacrolimus.

## NK-cell activation models mediated by the interaction between KIR and its ligand

KIR is one of the major receptors involved in regulating the activity and differentiation of NK cells (Leung, 2011; Dubreuil et al., 2021) There are two major subtypes of KIR—stimulatory and inhibitory receptors. Some KIRs can recognize HLA class I as a ligand. Among these KIRs, KIR2DL1/2DS1, KIR2DL2/3, KIR3DL1, and KIR3DL2 have been well examined (Leung, 2011). Although there are a few exceptions, KIR2DL1/2DS1 recognizes HLA-C with Lys80 (group C2) and KIR2DL2/3 recognizes HLA-C with Asp80 (group C1). Similarly, KIR3DL1 recognizes HLA-Bw4 and KIR3DL2 recognizes HLA-A3/A11. In certain settings of HLA-mismatched HSCT, interactions between KIR and its ligand can be altered, resulting in the activation of NK cells.

The effect of KIR-mediated NK-cell activation on HSCT outcomes was first examined by Ruggeri et al. (2007), Ruggeri et al. (2002), and Ruggeri et al. (1999). In their haploidentical transplantation analysis, they demonstrated that the presence of KIR2DL1, KIR2DL2/3, and KIR3DL1 ligand mismatch (HLA-C1, C2, and Bw4 mismatch) between the donor and recipient could result in the loss of KIR-mediated NK-cell suppression and could activate NK-cell function, which improves HSCT outcomes. Furthermore, they also examined the effects in mouse models and showed that the involvement of NK cells in reducing disease relapse, GVHD, and in improving engraftment had favorable effects on HSCT (Ruggeri et al., 2002). The KIR—ligand mismatch model was then verified in various settings of the HSCT cohort. However, the results were controversial and the favorable effects were suggested to be predominantly associated with T-cell depleted haploidentical transplantation (Giebel et al., 2003; Farag et al., 2006; Morishima et al., 2007; Sun et al., 2007).

Other than the KIR–ligand mismatch model, which does not consider the donor KIR genotype, some NK-cell activation models mediated by KIR have also been proposed. The receptor—ligand model considers the matching between the donor inhibitory KIR genotype and the recipient KIR ligand type (Leung et al., 2004; Hsu et al., 2005). Hsu et al. (2005) analyzed the T-cell depleted BMT cases with the receptor–ligand model and found that patients who did not have one or more ligand for donor KIR showed superior disease free and overall survival. Similar to this receptor–ligand model, a missing-ligand model that only considers the recipient’s KIR-ligand interaction was also reported. If the patient’s KIR-ligand group is homozygous, such as HLA-C1/C1 or C2/C2, inhibitory KIR without the ligand would facilitate NK-cell activation in contrast to the heterozygous group (Hsu et al., 2006; Miller et al., 2007; Arima et al., 2018).

There is another well-tested model that considers the presence of the donor’s stimulatory KIR. The KIR gene cluster is located on chromosome 19 and its haplotypes are classified into two major groups (Leung, 2011; Dubreuil et al., 2021). Haplotype group A have only one stimulatory KIR, KIR2DS4, in addition to the inhibitory KIRs, whereas group B have several other stimulatory KIRs. This suggests that donors with the group B haplotype (A/B or B/B) are more susceptible to NK-cell activation than donors with only the group A haplotype (A/A). The benefit of the KIR haplotype B donor was first demonstrated by Cooley et al. (2009). They analyzed myeloablative HLA-matched BMT/PBSCT and found improved relapse-free survival rates in patients undergoing transplantation with the KIR B haplotype donor. The effect of this KIR B haplotype has also been examined in other studies (Cooley et al., 2010; Zhou et al., 2014) Recently, the KIR-mediated NK-cell activation model was evaluated, taking into account genetic polymorphisms of KIR and HLA (Gagne et al., 2009; Boudreau et al., 2017; Shaffer et al., 2021).

## Effects of KIR–ligand mismatch on CBT

In CBT, the early recovery of NK cells and their long-term sustained increase has been reported, along with delayed recovery of T-cell function (Jacobson et al., 2012; Kanda et al., 2012; Bejanyan et al., 2018; Hattori et al., 2018; Ando et al., 2020). Based on these findings, theoretically, it is assumed that NK-cell function may be more clearly detected in CBT than in BMT/PBSCT. As of date, the effects of KIR–ligand interaction models in CBT have been examined in several studies, albeit not sufficiently, compared with those in BMT/PBSCT (Brunstein et al., 2009; Willemze et al., 2009; Garfall et al., 2013; Tanaka et al., 2013; Rettman et al., 2016; Rocha et al., 2016; Sekine et al., 2016; Martínez-Losada et al., 2017). As shown in Table 2, some results suggest an improvement in CBT outcomes (Willemze et al., 2009), whereas others show no effect (Tanaka et al., 2013) or increases in the nonrelapse mortality (NRM) (Brunstein et al., 2009). The reason for the inconsistency is unknown; however, it may be attributed to the differences in patient age, disease type, or GVHD prophylaxis. It has been reported that the frequency of viral infection, such as with cytomegalovirus (CMV), is different between children and adults (Yokoyama et al., 2019), which may cause the inconsistency in results. In addition, more distinctive effect of KIR–ligand mismatch has been shown in AML than in acute lymphoblastic leukemia (Ruggeri et al., 2002). Furthermore, because T- and NK-cell functions are known to be antagonistic (Lowe et al., 2003; Cooley et al., 2005), the use of antithymocyte globulin (ATG) for GVHD prophylaxis, that is, *in-vivo* T-cell depletion, can indirectly affect the NK-cell activity. In addition, the use of mycophenolate mofetil (MMF) for GVHD prophylaxis may cause a weak T-cell suppression compared with that cause by methotrexate (MTX) (Terakura et al., 2017a; 2017b), whereas it elicits a stronger inhibitory effect on the NK-cell function (Ohata et al., 2011). Another issue that may impact the KIR–ligand interaction effect in CBT is the method for determining the KIR–ligand mismatch. Because activation intensity and rate of expression along NK-cell maturation are different for each KIR (Foley et al., 2012a; Nguyen et al., 2017), it would be preferable to examine each KIR subtype mismatch individually rather than to analyze them as a bulk.

**TABLE 2:** Cord blood transplantation (CBT) outcomes resulting from the KIR–ligand effect.

References	N	Age	Disease	CBT year	Double cord	KIR mismatch model	Intensity of conditioning	ATG	GVHD prophylaxis	Effect of KIR-mediated mismatch on CBT outcomes
Willemze et al., 2009, Leukemia; 23 (3): 492–500	218	13.8 (0.5–69)	AML 43% ALL 57%	1997–2007	none	Ligand–ligand	MAC: 83% RIC: 17%	81%	CSA alone: 11%CSA/Steroid: 63%CSA/MMF: 16% Other: 10%	CBT with KIR—L mismatch showed improved LFS (HR 2.05, *p* = 0.0016) and OS (HR 2.0, *p* = 0.004)
Brunstein et al., 2009, Blood. 113 (22): 5,628–34	*MAC* 155 *RIC* 102	*MAC* KIR–L mm: 15 (0.6–53) KIR–L match: 15.9 (1–59) *RIC* KIR–L mm: 48 (22–69) KIR–L match: 52 (6–68)	*MAC* AML: 39% *RIC* AML: 27%	1998–2006	*MAC* 41% *RIC* 0%	Ligand–ligand	NA	35%	*MAC*CSA/Steroid: 39%CSA/MMF: 61% *RIC*CSA/MMF: 100%	In CBT with RIC, KIR—L mismatch was associated with the development of severe aGVHD (RR 1.8, *p* = 0.02) and increased risk of death (RR 1.8, *p* = 0.05)
Tanaka et al., 2013, Blood Cancer J; 3 (11): e164	643	ALL KIR–L match: 27 KIR-L mm: 33 AML KIR–L match: 47 KIR–L mm: 50	AML 56% ALL 55%	2001–2010	none	Ligand–ligand	MAC: 71% RIC: 29%	none	CSA ± MTX TAC ± MTX	No effects of the KIR—L mismatch
Garfall et al., 2013, Bone Marrow Transplant. 48 (7): 1,000–2	80	48 (21–67)	AML: 38.8% MDS: 5% CML: 2.5%	2003–2010	100%	Ligand–ligand	MAC: 26.2% RIC: 73.8%	73.8%	TAC/Sirolimus 67.5%TAC or CSA/MMF 32.5%	No effects of the KIR—L mismatch
Rocha et al., 2016; Biol Blood Marrow Transplant. 22 (7): 1,284–1,289	*HLA 6–7/8 matched* 199 *HLA 3–5/8 matched* 212	*HLA 6–7/8 matched* over 16 yo: 28% *HLA 3–5/8 matched* over 16 yo: 29%	AML 100%	2000–2010	none	Ligand–ligand	MAC: 100%	*HLA 6–7/8 matched* 70% *HLA 3–5/8 matched* 92%	CSA or TAC based	*HLA 6–7/8 matched* No effects of the KIR—L mismatch *HLA 3–5/8 matched* KIR–ligand mismatch in the HVG direction was associated with NRM (HR 2.26, *p* = 0.008) and OS (HR 1.78; *p* = 0.008)
Sekine et al., 2016; Blood. 128 (2): 297–312	*Discovery cohort* 110 *Validation cohort* 94	*Discovery cohort* 38 (2–73) *Validation cohort* 41 (1–73)	*Discovery cohort* AML: 40% *Validation cohort* AML: 41%	2009–2012	*Discovery cohort* 95% *Validation cohort* 94%	Receptor–ligand	*Discovery cohort* MAC: 72% RIC: 28% *Validation cohort* MAC: 78% RIC: 22%	NA	NA	Patients homozygous for the HLA—C2 group had a higher 1-year relapse rate and worse survival after CBT than others. HLA—C1/x patients receiving a CB graft with both the HLA–C1 and KIR2DL2/L3/S2 genotypes had lower 1-year relapse rates (HR 6.98, *p* = 0.002) and superior survival (HR 3.31, *p* = 0.003). HLA-C2/C2 patients had lower relapse and better OS if they received a graft with both the HLA—C2 and KIR2DL1/S1 genotypes
Rettman et al., 2016; Bone Marrow Transplant. 51 (11): 1,499–1,503	227	49.5 (4.3–69.1)	Myeloid: 50.7% Lymphoid 46.3%	2005–2011	100%	Receptor–ligand	MAC: 27% RIC: 73%	13.2%	CSA/MMF: 88.1%	*Better neutrophil recovery:* winner CB graft: KIR2DL2/L3-positive, patient: HLA—C1-negative winner CB graft: KIR2DS1-positive, loser CB graft: HLA—C2-positive *Increased incidence of aGVHD:* winner CB graft: KIR2DL2/L3-positive, patient: HLA—C1-positive *Decreased incidence of aGVHD*: winner CB graft: KIR3DS1-positive, loser CB graft: HLA—Bw4-positive, and the combination loser CB graft: KIR2DL1-positive, patient: HLA—C2-positive *Increased incidence of cGVHD*: winner CB graft: KIR2DL2/2DL3-positive, loser CB graft: HLA—C1-positive *Increased NRM*: winner CB graft: KIR2DL2/L3-positive, loser CB graft: HLA—C1-positive
Martínez-Losada et al., 2017; Front Immunol. 8:810	33	11 (1–48)	AML: 39% ALL: 55% Others: 6%	2002–2011	none	Ligand–ligand	MAC: 88% RIC: 12%	100%	CSA/Steroid: 76%CSA/MMF: 24%	CBT with HLA–C1/C1 or—C2/C2 showed a lower relapse than patients with HLA—C1/C2 (RR 3.75, *p* = 0.03)
Yokoyama et al., 2021; Bone Marrow Transplant. 56 (6): 1,352–1,363	2,502	*KIR–L mm* No CMV: 49 (17–73) CMV: 56 (16–72) *w/o KIR–L mm* No CMV: 50 (16–77) CMV: 55 (16–77)	AML: 63% ALL: 20% MDS: 15% CML: 3%	2007–2018	none	Ligand–ligand	MAC: 68% RIC: 32%	none	CNI/MTX: 60% CNI/MMF: 31% Others: 8%	In the presence of HLA—Bw4 or —A3/A11 mismatch (KIR3DL–ligand mismatch), CMV reactivation up to 100 days had a favorable effect on relapse (HR 0.54, *p* = 0.032). In the presence of other KIR—ligand mismatch types, this effect was not noted
Yokoyama et al., 2021; Bone Marrow Transplant. 56 (12): 3,059–3,067	3,010	*TAC/MTX* w/o KIR–L mm: 52 (16–80) KIR–L mm: 52 (16–71)*TAC/MMF* w/o KIR–L mm: 60 (16–80) KIR–L mm: 60.5 (19–77)	AML: 65% ALL: 19% MDS: 14% CML: 3%	2008–2018	none	Ligand–ligand	MAC: 66% RIC: 34%	none	TAC/MTX: 55%TAC/MMF: 45%	HLA—C2 mismatch had a favorable effect on relapse (HR, 0.56, *p* = 0.0043) and OS (HR, 0.72, *p* = 0.037) only in CBT with GVHD prophylaxis using MTX. In the CBT using MMF for GVHD prophylaxis, HLA—A3/A11 mismatch worsened NRM (HR, 1.93, *p* < 0.001) and OS (HR, 1.48, *p* = 0.014)
Yokoyama et al., 2022; Bone Marrow Transplant. 57 (7): 1,171–1,179	2,299	*CNI/MTX* w/o HLA–C2 mm: 50 (16–79) HLA–C2 mm: 53 (16–70)*CNI/MMF* w/o HLA–C2 mm: 61 (16–80) HLA–C2 mm: 59 (17–77)	AML: 100%	1998–2019	none	Ligand–ligand	MAC: 72% RIC: 28%	none	CNI/MTXCNI/MMF	HLA—C2 mismatched CBT using CNI/MTX for GVHD prophylaxis had a favorable effect on relapse (HR 0.61, *p* = 0.017) and OS (HR 0.72, *p* = 0.016). The favorable KIR—ligand mismatch effect was not observed in CBT with the KIR—ligand mismatch types other than HLA—C2 and those using CNI/MMF for GVHD prophylaxis
Kawahara et al., 2022; Transplant Cell Ther. 28 (9): 598.e1-598.e8	91	10 (0–19)	T-ALL: 100%	1999–2017	none	Ligand–ligand	MAC: 76% RIC: 24%	3%	CSA-based: 31% TAC-based: 67% Others: 2%	KIR—ligand mismatch in the GVH direction was associated with a significant reduction in relapse rate (HR 0.19, *p* = 0.002), resulting in better LFS (HR 0.18, *p* = 0.010) and OS (HR 0.26, *p* = 0.048) without increasing NRM.
Iemura et al., 2022; Bone Marrow Transplant. 57 (5): 781–789	429	55 (16–77)	AML: 45% MDS: 13% ALL: 15% ML: 22 Others: 6%	2003–2019	none	Ligand–ligand	MAC: 55% RIC: 45%	NA	CNI alone: 7%CNI/MTX: 30%CNI/MMF: 63%CNI/MTX/MMF: 0.2%	Patients harboring both HLA ≥3 allele mismatches and inhibitory KIR—ligand mismatches had a significantly greater prevalence of viral infection (HR 1.66, *p* = 0.04) and had poorer outcomes in terms of NRM (HR 1.61, *p* = 0.05)
Otegbeye et al., 2022; Transplant Cell Ther. 28 (8): 483.e1-483.e7	*Single cord* 900 *Double cord* 954	*Single cord* 8 (0–81) *Double cord* 34 (1–70)	*Single cord* AML: 44% *Double cord* AML: 52%	2008–2017	NA	Ligand–ligand	MAC: 100%	*Single cord* 43% *Double cord* 14%	*Single cord*CNI/MTX: 12% CNI/others: 88% *Double cord*CNI/MTX: 3% CNI/others: 97%	In single unit CBT, HVG or bidirectional KIR–ligand mismatch was initially associated with higher NRM in the first 6 months after transplantation, but this effect was nullified after 6 months. In double unit CBT, no significant differences were seen in engraftment, OS, or NRM.

N, number of patients; MAC, myeloablative conditioning; KIR–L mm, killer-immunoglobulin like receptor–ligand mismatch; RIC, reduced-intensity conditioning; ALL, acute lymphoblastic lymphoma; AML, acute myeloid leukemia; CMV, cytomegalovirus; TAC, tacrolimus; MTX, methotrexate; w/o, without; MMF, mycophenolate mofetil; CNI, calcineurin inhibitor; MDS, myelodysplastic syndrome; CML, chronic myeloid leukemia; ML, malignant lymphoma; ATG, antithymocyte globulin; CSA, cyclosporin A; LFS, leukemia-free survival; HR, hazard rate; OS, overall survival; aGVHD, acute graft-versus-host disease; RR, relative risk; *p*, *p*-value; HVG, host-versus-graft; NRM, nonrelapse mortality.

To overcome these problems in the analysis of the KIR–ligand mismatch effect, a study with the largest number of CBT cases was conducted (Yokoyama et al., 2021a). A total of 3,010 cases of adult single unit CBT were classified based on GVHD prophylaxis (tacrolimus/MTX or tacrolimus/MMF), and the effects of the KIR2DL1-, KIR3DL1-, and KIR3DL2–ligand mismatches (HLA-C2, Bw4, and A3/A11 mismatches, respectively) were examined (Table 2). The HLA–C2 mismatch had a favorable effect on relapse [hazard ratio (HR), 0.56, *p* = 0.0043] and OS (HR, 0.72, *p* = 0.037) only in the CBT, with GVHD prophylaxis using MTX, whereas these effects were not noted in the other types of KIR–ligand mismatch. In the CBT using MMF for GVHD prophylaxis, the HLA–A3/A11 mismatch had a negative effect on the NRM (HR, 1.93, *p* < 0.001) and OS (HR, 1.48, *p* = 0.014). After further accumulation of cases, we conducted a similar analysis focusing on 3,627 patients with AML and found that only in the group using MTX for GVHD prophylaxis, the presence of HLA–C2 mismatch resulted in favorable effects, such as reduced incidence of relapse (HR 0.61, *p* = 0.017) and improved OS (HR 0.72, *p* = 0.016) (Yokoyama et al., 2022).

## NK-cell maturation induced by CMV reactivation after HSCT and the effects of the KIR–ligand mismatch

The percentage of mature NK cells has been shown to rapidly increase after CMV reactivation in various settings of HSCT (Foley et al., 2012b, 2012a; Li et al., 2019; Rashidi et al., 2019; Zaghi et al., 2021). Most of these cells have CD56 dim, NKG2A-negative, KIR-positive, and NKG2C-positive phenotypes, and are classified as memory-like NK cells (Foley et al., 2012b). CMV reactivation has been suggested to reduce AML relapse (Elmaagacli et al., 2011; Green et al., 2013; Manjappa et al., 2014; Jang et al., 2015; Takenaka et al., 2015; Ramanathan et al., 2016; Teira et al., 2016; Yokoyama et al., 2020), and the involvement of these mature NK cells has been postulated as a possible underlying mechanism. Despite some negative reports (Takenaka et al., 2015; Teira et al., 2016) and controversial results, the cooperative effects of KIR–ligand mismatch in reducing AML relapse by CMV reactivation have been reported (Yokoyama et al., 2021b). It was reported that in the presence of the HLA—Bw4 or—A3/A11 mismatch (KIR3DL—ligand mismatch), CMV reactivation for up to 100 days had a favorable effect on the relapse rate (HR 0.54, *p* = 0.032), whereas in the presence of other KIR–ligand mismatch types, these effects were not noted.

## Conclusion

The effects of KIR–ligand interactions on NK cells in CBT remain unclear. This might be partially due to the difficulty in demonstrating the complex mechanisms of NK-cell activation in a model that only considers a single receptor, such as KIR. It might, therefore, be necessary to make a model that can consider multiple mechanisms of NK-cell activation, or to analyze a homogeneous population extracted from many cases. Despite such complex situations, attempts are being made to construct a more reliable model of NK-cell activation (Dhuyser et al., 2022). These findings will not only be useful for donor selection in CBT, but would also be useful for adoptive NK-cell therapy to maximize their efficacy in the future.
